# Resistance Mutation R292K Is Induced in Influenza A(H6N2) Virus by Exposure of Infected Mallards to Low Levels of Oseltamivir

**DOI:** 10.1371/journal.pone.0071230

**Published:** 2013-08-12

**Authors:** Anna Gillman, Shaman Muradrasoli, Hanna Söderström, Johan Nordh, Caroline Bröjer, Richard H. Lindberg, Neus Latorre-Margalef, Jonas Waldenström, Björn Olsen, Josef D. Järhult

**Affiliations:** 1 Section of Infectious Diseases, Department of Medical Sciences, Uppsala University, Uppsala, Sweden; 2 Section of Bacteriology and Food Safety, Department of Biomedical Sciences and Veterinary Public Health, Swedish University of Agricultural Sciences, Uppsala, Sweden; 3 Department of Chemistry, Umeå University, Umeå, Sweden; 4 Section of Pathology, Pharmacology and Toxicology, Department of Biomedical Sciences and Veterinary Public Health, Swedish University of Agricultural Sciences, Uppsala, Sweden; 5 Section for Zoonotic Ecology and Epidemiology, School of Natural Sciences, Linnaeus University, Kalmar, Sweden; National Institute for Viral Disease Control and Prevention, CDC, China, China

## Abstract

Resistance to neuraminidase inhibitors (NAIs) is problematic as these drugs constitute the major treatment option for severe influenza. Extensive use of the NAI oseltamivir (Tamiflu®) results in up to 865 ng/L of its active metabolite oseltamivir carboxylate (OC) in river water. There one of the natural reservoirs of influenza A, dabbling ducks, can be exposed. We previously demonstrated that an influenza A(H1N1) virus in mallards (*Anas platyrhynchos*) exposed to 1 µg/L of OC developed oseltamivir resistance through the mutation H274Y (N2-numbering). In this study, we assessed the resistance development in an A(H6N2) virus, which belongs to the phylogenetic N2 group of neuraminidases with distinct functional and resistance characteristics. Mallards were infected with A(H6N2) while exposed to 120 ng/L, 1.2 µg/L or 12 µg/L of OC in their sole water source. After 4 days with 12 µg/L of OC exposure, the resistance mutation R292K emerged and then persisted. Drug sensitivity was decreased ≈13,000-fold for OC and ≈7.8-fold for zanamivir. Viral shedding was similar when comparing R292K and wild-type virus indicating sustained replication and transmission. Reduced neuraminidase activity and decrease in recovered virus after propagation in embryonated hen eggs was observed in R292K viruses. The initial, but not the later R292K isolates reverted to wild-type during egg-propagation, suggesting a stabilization of the mutation, possibly through additional mutations in the neuraminidase (D113N or D141N) or hemagglutinin (E216K). Our results indicate a risk for OC resistance development also in a N2 group influenza virus and that exposure to one NAI can result in a decreased sensitivity to other NAIs as well. If established in influenza viruses circulating among wild birds, the resistance could spread to humans via re-assortment or direct transmission. This could potentially cause an oseltamivir-resistant pandemic; a serious health concern as preparedness plans rely heavily on oseltamivir before vaccines can be mass-produced.

## Introduction

Resistance to the antiviral drugs neuraminidase inhibitors (NAIs) is a problem as they are the best available option for treatment and prophylaxis of influenza A virus infection. The NAI oseltamivir (Tamiflu®) has been stockpiled in large quantities in many nations as part of preparedness plans for a new influenza pandemic [1], [2]. The use of oseltamivir is especially important in the first phase of a pandemic, before vaccines can be mass-produced. Thus, a new pandemic strain resistant to oseltamivir would be of substantial individual and public health concern. The emergence and spread of the resistant seasonal (pre-pandemic) A(H1N1) strain 2007–2009 tilted the previous concept of decreased fitness of resistant viruses [3]. If a resistance mutation occurs in a permissive genetic background the decreased fitness can be compensated for [4], [5]. In wetland birds, the natural reservoir for influenza A virus, the genetic variability of influenza A virus is tremendous; 16 haemagglutin (HA) and 9 neuraminidase (NA) surface proteins exist in varying combinations [6], [7]. All studied pandemics (from the last century) have contained gene segments from avian influenza A virus lineages [7]–[10] and thus there is good reason to believe that this will be the case also in future pandemics.

Oseltamivir administered orally (as the pro-drug oseltamivir phosphate) is readily absorbed and converted to the active metabolite oseltamivir carboxylate (OC). At least 75% of a given dose reaches the blood circulation as OC and is then excreted unchanged via the urine. OC is stable in sewage treatment processes and has been detected in effluents from sewage treatment plants (up to 1.21 µg/L) and in river water (up to 865 ng/L) [11]–[15]. Sampling in Germany suggests discharge from pharmaceutical industries as another contributing source [16].

There are two phylogenetic groups of neuraminidases (NAs), N1 (including N1, N4, N5, N8) and N2 (including N2, N3, N6, N7, N9). Resistance mutations and the exact binding site of OC adjacent to the active site differ between the two groups. The most common resistance mutations are H274Y (N2 numbering, this numbering is used throughout the paper) in the N1, and R292K or E119V in the N2 group [17]–[20].

There is an interdependence of HA and NA activity for optimal viral replication and NAIs can induce mutations in HA as well as in NA residues [21]. Once a NAI resistance mutation has occurred, compensation of decreased NA function by new compensatory mutations have been described in both HA and NA. In N1 virus compensatory mutations in NA [4], [22] and concomitant mutations at the receptor binding site in HA[23]–[25] related to H274Y have been described. In N2 virus with the R292K mutation no compensatory mutations in NA have been defined, however secondary balancing mutations in the HA are described; in a patient (R228S) [26] and in vitro (N199S and G143E) [27].

**Figure 1:**
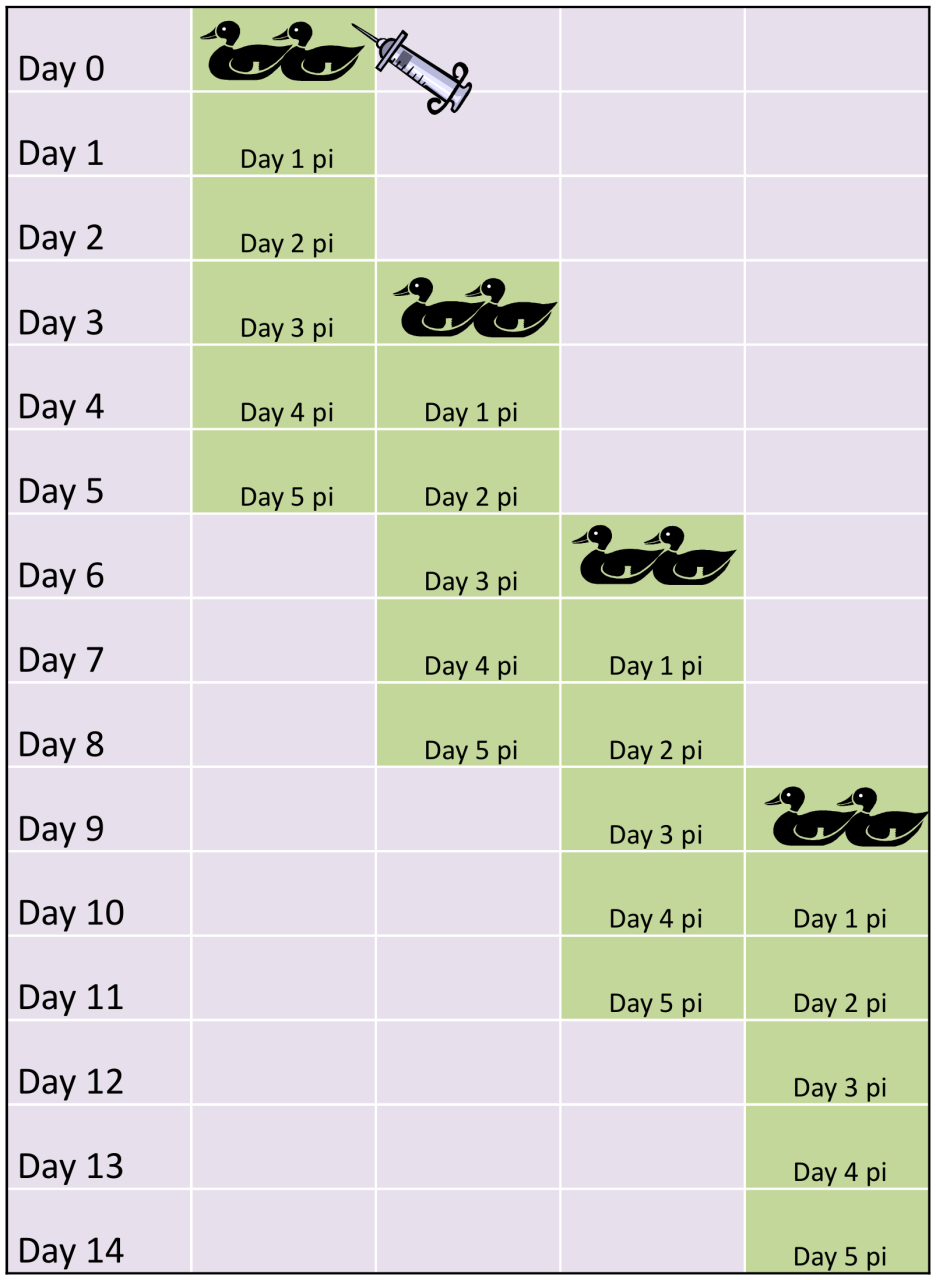
Set-up of the Mallard Model. Each experiment consisted of 4 generations of mallards with 2 individuals in each. The first generation of mallards was infected by esophageal inoculation with the A/*Mallard*/Sweden/50908/2006(H6N2) virus (day 0) and were put in the experimental room where the sole water source contained OC. The following generations were introduced with 3-day intervals allowing the infection to be fecal-orally transmitted between generations during two days. Five days after introduction, each generation birds were removed and euthanized. The water in the sole water source of the birds was changed daily and contained the same level of OC during each 14 day-experiment. Three subsequent experiments were performed with OC levels of 120 ng/L, 1.2 µg/L and 12 µg/L.

We previously found that a low-pathogenic avian influenza A(H1N1) virus developed resistance to oseltamivir when infected mallards were exposed to low, environmental-like levels of OC (1 µg/L) [28]. It is likely that resistance can be induced in all influenza A viruses with the N1 group of NAs under these circumstances. However, given the distinct characteristics of the N2 group of NAs, it is unclear if resistance can be induced also on this phylogenetic group of influenza A viruses under conditions approximating an environmental situation. To test this, we used the same mallard model and an influenza A(H6N2) virus isolated from a wild mallard in Sweden. In brief, we inoculated two mallards with the virus and then successively introduced new generations of birds in three subsequent experiments with increasing concentrations of OC (120 ng/L, 1.2 µg/L or 12 µg/L) in the ducks’ sole water source. We then analyzed virus from fecal samples with regard to resistance mutations and sensitivity to NAIs (Figure 1, details in *Materials and Methods*).

## Results

### Infection of Mallards

**Figure 2:**
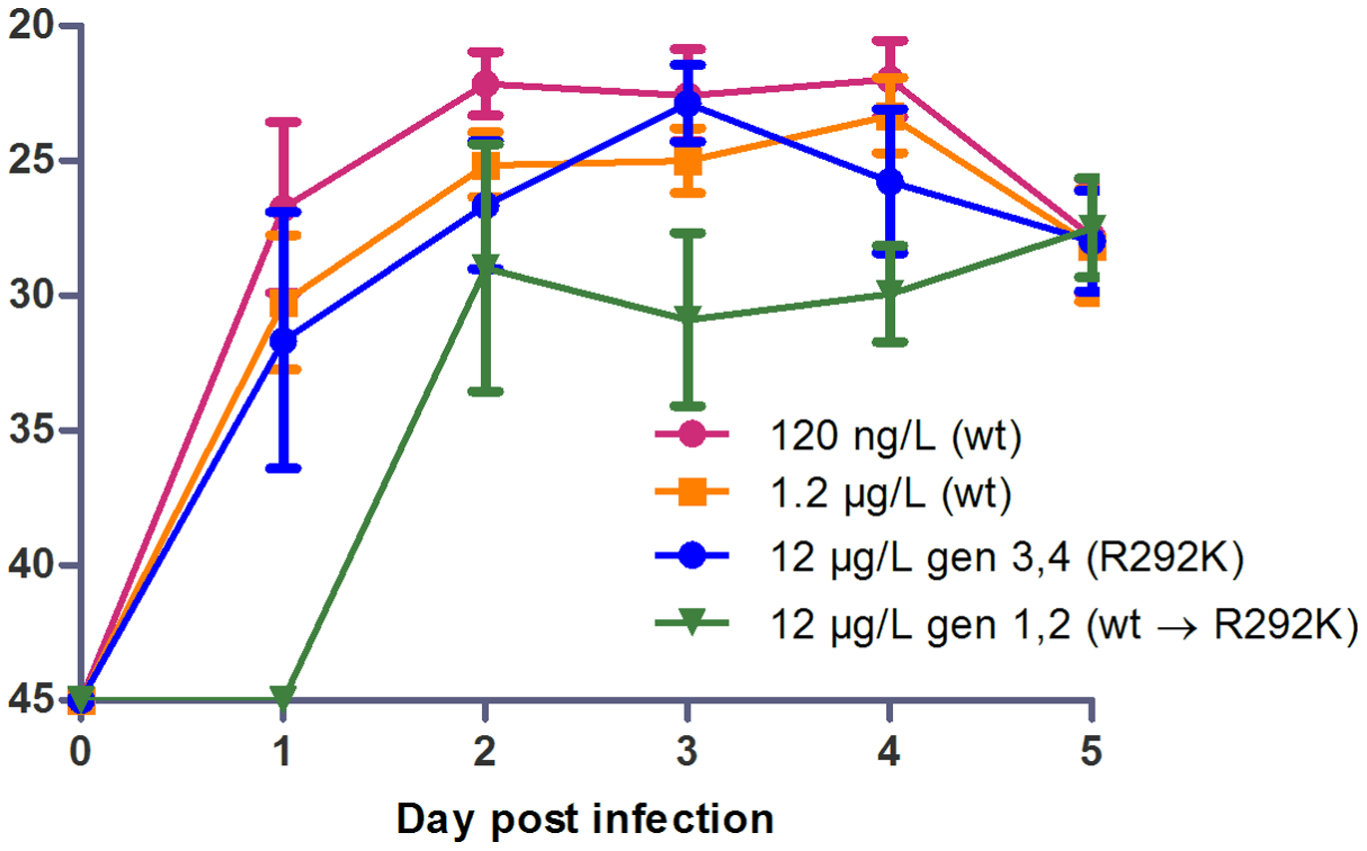
Viral Shedding. RRT-PCR performed on fecal samples. The Y-axis displays CT-values. The cut-off for negative results was set to a CT-value of 45. Error bars display ± standard deviation. n = 8 in 120 ng/L and 1.2 µg/L groups, n = 4 in the two groups from the 12 µg/L experiment.

No mallards were infected with influenza A virus prior to entering the animal house facilities, verified by serology and real time reverse transcriptase (RRT-) PCR. The exposure to OC started at the time of inoculation and lasted all through the experiment. From the birds exposed to 120 ng/L and 1.2 µg/L of OC viral shedding occurred from day one post infection (pi) through day five, with a maximum at day 2–4 pi and a slight decrease the last day of each generation (Figure 2). No difference in shedding was seen when comparing the birds infected by esophageal inoculation to those infected by transmission from other birds (data not shown), consistent with our previous study [29]. Wild-type virus was present in the two first generations of the 12 µg/L experiment; in those generations no virus was shed day 1 pi and viral shedding seemed to be lower at day 3–4 pi compared to the 120 ng/L and 1.2 µg/L experiments. The two later generations of the 12 µg/L experiment, in which the virus was R292K mutated, had similar shedding patterns as those seen at OC levels of 120 ng/L and 1.2 µg/L (Figure 2).

Throughout all experiments, influenza A virus was detected in the water by RRT-PCR. CT-values ranged from 22 to 32 and were similar in all three experiments with exception of day 1 pi for the 1.2 µg/L and the 12 µg/L experiments where CT values of 36.5 were found, indicating lower viral titers.

### Analysis of Viral Mutations in the Neuraminidase (NA) and Hemagglutinin (HA)

No mutations related to resistance were seen when sequencing NA in fecal samples from ducks exposed to OC levels of 120 ng/L or 1.2 µg/L. At 12 µg/L of OC, the resistance mutation R292K (the arginine codon AGA changed to the lysine codon AAA) in the NA occurred in the first generation of mallards 4 days pi as a mixed genotype, and dominated from day 5 pi and throughout the experiment. Sequencing of NA from water samples revealed the R292K mutation from day 5 pi and onwards.

In addition to the R292K mutation, the D113N and D141N mutations evolved (the aspartic acid codon GAC changed to the asparagine codon AAC) in the 12 µg/L experiment from day 7 and 8 pi respectively. NA sequencing of the fecal samples showed either D113N or D141N, or mixed sequencing results of both mutations, but both mutations did not dominate at the same time. The D113N mutation was found in water samples from day 8 pi and D141N from day 12 pi, whereafter both mutations were present as mixed genotypes. Taken together, these results suggest that either of the mutations D113N or D141N was present in a single virus, but not both mutations at the same time.

Sequencing of HA of 6 isolates from the 12 µg/L experiment revealed the E216K mutation (H3-numbering, the GAA codon for glutamic acid changed to AAA for lysine) in the same isolates that also harbored R292K in combination with D113N or D141N in the NA (but not in wild type isolates or isolates with only R292K), i.e. E216K was present in the latest samples from the experiment.

### Propagation in Embryonated Hen Eggs

**Figure 3:**
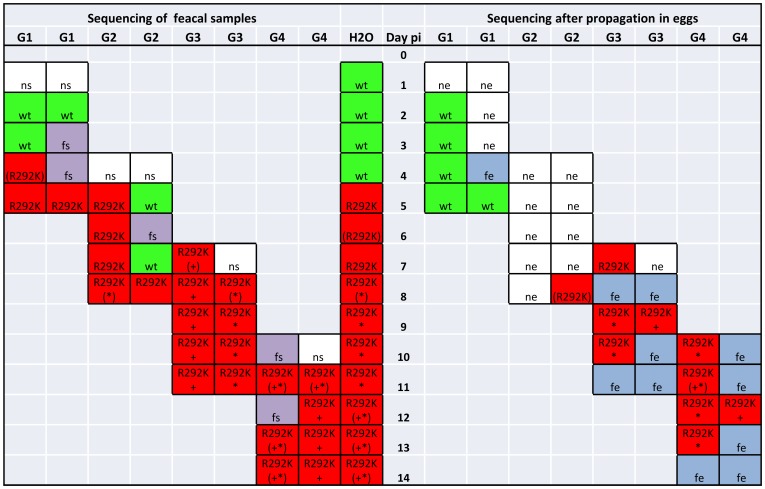
Mutation Analysis of NA the 12 µg/L Experiment. Sequencing of the NA gene from fecal samples, water and post egg propagation from the 12 µg/L experiment. G = generation. Pi = post infection. H2O = water. Green wt = wild type. Red R292K = mutated at 292. Red (R292K) = mixed genotype R292 and K292. * = D113N. (*) = mixed genotype D113 and N113.**+** = D141N. (**+**) = mixed genotype D141 and N141. Purple fs = failure to sequence. Blue fe = failure to propagate in embyonated hen eggs. White ns = not sequenced. White ne = not propagated in embryonated hen eggs.

Prior to phenotypic analysis of resistance the viral samples were propagated in specific pathogen free embryonated hen eggs. In total, virus from 38 fecal samples was processed for egg propagation. Of those, 13 of 23 isolates (56%) originally carrying R292K were successfully propagated and 13 of 15 isolates (87%) with wild-type R292. When re-sequencing the isolates after the propagation process we found wild-type R292 in 3 of the 13 R292K isolates, all from the first generation of birds of the 12 µg/L experiment, and in one isolate from the second generation birds we found mixed genotype after the propagation. The 9 following R292K isolates from generation 2, 3 and 4 retained the mutation through the propagation process. All 9 isolates additionally had the D113N or D141N mutations that also were retained through the egg propagation (Figure 3). The E216K mutation in HA also persisted through the egg propagation process.

### Neuraminidase Activity and Inhibition by Oseltamivir and Zanamivir

Inhibition of NA activity with OC and zanamivir (ZA) was compared between isolates with the R292K-mutation (10 evaluable isolates) and wild-type isolates (5 from 120 ng/L, 4 from 1.2 µg/L and 5 from 12 µg/L OC-levels). The mean 50% inhibitory concentration (IC_50_) for OC was 2,900 nM (95% CI of median 2,600, 3,000) of the R292K isolates, significantly different at a 95% level to 0.21 nM (95% CI of median 0.15, 0.21) of the wild type isolates (*P* = 0.000082), corresponding to a 13,000 fold reduced sensitivity (highly reduced according to WHO criteria [30]). Mean IC_50_ for ZA was 5.3 nM (95% CI of median 4.5, 6.8) of R292K isolates and 0.68 (95% CI of median 0.44, 0.92) nM of wild type isolates, as well significantly different at a 95% level (*P* = 0.000082), corresponding to a 7.6 fold reduced sensitivity (normal according to WHO criteria [30]). The IC_50_ values for wild type genotype in the experiments were similar to the inoculate strain A/*Mallard*/Sweden/50908/2006(H6N2) which had an IC_50_ of 0.26 nM for OC (95% CI of median 0.093, 0.60) and 0.55 nM for ZA (95% CI of median 0.41, 0.69) in 8 repeated assays (Table 1).

**Table 1:** Viral Sensitivity to Oseltamivir and Zanamivir by IC_50._

Virus	IC_50_ OC	IC_50_ ZA
	nM (95% CI_median_)	nM (95% CI_median_)
50908 wt	0.26*	0.55*
R292 (n = 14)	0.21 (0.15, 0.21)	0.68 (0.44, 0.92)
R292K (n = 9)	2,900 (2,600, 3,000)	5.3 (4.5, 6.8)

* – The IC_50_s of the original wild-type A/*Mallard*/Sweden/50908/2006(H6N2) virus (50908 wt) are means based on 8 repeated assays.

One R292K-isolate from day 9 pi had an IC_50_ of 770 nM for OC and 0.32 nM for ZA. We did not find a good explanation for this outlier; no additional mutations in the NA could be detected. This isolate has been excluded from the data presentation but the general conclusions of the study would not be affected if it had been included.

While assaying the NA activity by the relative fluorescence measurement prior to dilution of samples for the inhibition assay, we noted an approximately 70% reduction of NA activity of the R292K isolates compared to wild type with mean RFU 8,000 of the mutant (95% CI of median 3,900, 9,000), significantly different at a 95% level to 27,000 of wild type virus (95% CI of median 24,000, 32,000) (P<0.000001). No further enzymological evaluations of the NA characteristics were performed.

### Histopathologic Evaluation

Two birds from each experiment (one artificially inoculated and one infected by transmission from another bird) were necropsied at 5 days pi and examined with histopathology and immunohistochemistry (IHC) staining of influenza A nucleoprotein. One of six examined birds was infected with the R292K-mutated virus and the rest with wild-type virus. The R292K-mutant infected bird (fecal-orally infected) and one wild-type infected bird (artificially inoculated, from the 1.2 µg/L experiment) had one section each of the intestine where weak IHC positivity was demonstrated in distal epithelial cells and monocytes. The remaining four birds were negative with regard to IHC staining. The histopathologic evaluation showed that several sections of intestine had mild to moderate amounts of heterophils in the lamina propria but there was no correlation between presence of heterophils and IHC-positivity.

### OC Concentration in the Water

The average daily OC concentrations (each measured after 24 h of exposure when the water was changed) were 121±19 ng/L, 1,238±266 ng/L and 12,095±3,095 ng/L (± standard deviation) in the respective experiments. In comparison, the differences in average (n = 3) daily OC concentration between freshly prepared water and in the water after 24 h experiment was maximum ±30%. These concentration differences are within the general variation of chemical analysis of pharmaceuticals and can be caused by differences in procedures of the OC-water preparation, water sampling, pre-treatment of samples, and sample analysis. The limit of quantification (LOQ) of OC was 1 ng/L, the linearity (R2) of the calibration curve was 1.0000 and the relative standard deviation (RSD) between pseudo-triplicates (triplicates taken from one collected sample) was in the range 4 to 26%.

### Mutation Screening of NCBI Influenza Virus Sequence Database

Amino acid sequences of the NA protein from 2514 avian influenza A/N2 viruses were downloaded from the NCBI Influenza Virus Sequence Database [31] and analyzed. No sequences contained R292K. Only two isolates had other amino acids at position 292; one had R292W (a H9N2 virus isolated from a sparrow in China 2006, ADC97091) and one had a 14 amino acid sequence exchange including position 292 (a H9N2 virus isolated from a duck in China 2001, ABG27052). D113N and D141N were frequent variants, D113N in approximately 1–10% and D141N in approximately 20–40% (varying proportions with different HA subtype combination) although the mutations did not occur simultaneously.

## Discussion

In this study, we demonstrate resistance development in an influenza A(H6N2) virus in mallards exposed to 12 µg/L of OC in their sole water source. The resistance was caused by acquisition of the mutation R292K in the NA. The drug sensitivity was ≈13,000-fold decreased for OC and ≈7.6-fold decreased for ZA which is in parity with previous findings [19], [32] (Table 1).

The occurrence and relevance of the R292K mutation has been described already during the development phase of oseltamivir both *in vitro* [33], [34] and in humans treated with the substance during clinical trials [35], [36]. Clinically the mutation has occurred in oseltamivir-treated patients leading to prolonged viral carriage [26], [37].

The difference in sensitivity decrease to OC and ZA caused by R292K can be explained by the protein structure of N2 NAs. R292 is located at the catalytic site and OC affinity requires binding of the hydrophobic pentyl ether side chain deep in the catalytic site, this is not required for the binding of ZA. In wild-type N2-OC complex formation, the E276 residue moves and becomes salt linked to R224; this opens up a hydrophobic pocket where the OC pentyl ether group binds. The exchange of arginin to lysin at position 292 results in tighter binding with E276, which inhibits the movement and the creation of the hydrophobic pocket, whereby the affinity for OC is reduced. ZA binding does not require a conformational change in the active site and the slight decrease in binding to the R292K mutant is due to a lower ability to penetrate the active site [27], [38].

The R292K mutation has previously been shown to decrease NA activity [19], [27], [32] and to decrease replication capacity in cell cultures and in embryonated hen eggs, although conflicting results have been found in replication studies [33]–[35], [39]. Studies in mammal models have previously shown reduced infectivity in mice and ferrets [34]–[36] and no or reduced transmissibility in ferrets [36], [39] of R292K-mutated virus. We evaluated viral replication in the mallard intestine and transmissibility between individuals by measuring viral shedding in fecal samples using RRT-PCR. No difference in shedding pattern was observed when comparing wild-type virus from the 120 ng/L and 1.2 µg/L OC levels and R292K-mutated virus from the 12 µg/L OC level suggesting a retained replicative capacity for the mutant. The unchanged viral shedding pattern of the two last generations of mallards in the 12 µg/L experiment (shedding R292K mutated virus) furthermore indicates that the transmissibility was not reduced for the mutant virus (Figure 2). The set-up of our model with close contact between birds in a confined space while shedding a maximum of virus may however not be optimal to detect a slight decrease in transmissibility. The two first generations mallards from the 12 µg/L OC level, during which the mutation developed, had lower viral excretions, especially the first days. It is probable that this was due to OC-inhibition of the replication of wild-type virus.

Although no proper kinetic assay of the NA enzyme was performed, we observed an approximately 70% reduction in NA-activity of the R292K isolates while preparing our viral samples for NAI inhibition analysis. These observations are in parity to what has previously been reported [19], [27], [32].

One of the birds from which sections of intestine were evaluated by IHC, was infected with R292K-mutated virus. The IHC stain was weakly positive in one section which is similar to what was seen in birds infected with wild-type virus, and to previous results from mallards infected with A(H1N1) wild-type virus in the same model [29]. This suggests similar infectivity of the R292K mutant compared to wild-type virus in the mallard gut. As the data regarding the mutated virus is from one bird only it must be interpreted with some caution. It does however demonstrate that fecal-oral infection with a R292K-mutated virus resulted in IHC positivity in the intestine 5 days pi which was not seen in any of the two birds fecal-orally infected with wild-type A(H6N2) virus. In our previous study, mallards infected with wild-type A(H1N1) virus showed IHC positivity in 29% of the birds [29].

We observed a decrease in recoverable virus after propagation in embryonated hen eggs when comparing R292K to wild-type, suggesting a decreased replication capacity in embryonated hen eggs. Among the mutants, the first 3 isolates had wild-type R292 and the fourth a mixed genotype when re-sequenced after the propagation process, while all later mutants retained the mutation. This might be caused by a higher proportion of wild-type virus being present in earlier samples despite the fact that the Sanger sequencing showed only AAA for lysine at the 292 position, as this method has a limited sensitivity, or it might reflect a stabilization of the mutation later in the experiment.

Our findings of additional mutations D113N or D141N in the NA and E216K in the HA together with R292K in the last isolates of the high-dose experiment, co-varied with the persistence of R292K through the egg propagation process. It is possible that the additional NA mutations altered the NA characteristics. Both D113N and D141N are frequently occurring variants in avian N2 strains and the residues are closely located to each other. The residues are not situated immediately adjacent to the active site, but further structural analysis and functional evaluation of the mutations has not yet been performed. The E216K substitution in HA has not been reported in relation to NAI-resistance mutations in the NA. The residue is however close to the 220 loop of the HA binding site [21] and residues essential for binding specificity [40], [41] and might possibly compensate for the R292K NA mutation. These findings need to be further examined.

Persistence of R292K without drug pressure has been shown in vitro [27] but not in vivo in a ferret model [42] or in a treated patient [26]. When the NCBI database was screened, no R292K-carrying avian influenza A/N2 virus was found. This could indicate instability of the mutation without drug pressure or reflect that OC drug pressure has not yet made a significant imprint among circulating avian N2 viruses.

Several studies have detected OC in the aquatic environment [12]–[15], and generally environmental levels in the magnitude of lower to mid ng/L have been found. Up to 865 ng/L of OC has been detected in river water which is 14-fold lower than the 12 µg/L of OC where we observed resistance development. We do however argue that there are reasons to take our results into environmental account as the threshold for resistance development could lie anywhere between 1.2 and 12 µg/L and as seen in our previous experiment with an A(H1N1) virus the resistance mutation (H274Y) was detected at 1 µg/L in only two samples [28]; a similar resistance development could have been missed in the present, shorter experiment. Furthermore, avian influenza A viruses have a large genetic variability, and their sensitivity to NAIs vary to a larger extent than the sensitivity of mammalian viruses [43]; thus there is probably a variety of resistance thresholds in influenza viruses circulating among wild birds and viruses with lower thresholds than our randomly chosen H6N2 virus may well exist. Finally, although the general magnitude of environmental OC levels has been established, there is still a possibility that higher OC levels could occur, e.g. in a severe pandemic or locally when oseltamivir prophylaxis is extensively used to blanket an outbreak.

Like previously studied influenza pandemics, also future ones can be expected to contain genetic material from avian strains e.g. through re-assortment [8], [9]. Human adaptation of highly-pathogenic avian influenza is another dramatic scenario highlighted by recent demonstrations of a low genetic barrier to airborne mammal-to-mammal transmission of highly-pathogenic avian H5N1 viruses [44]–[46]. The switch of HA binding preference from sialic acid-2,3α-galactose to sialic acid-2,6α-galactose involved in this adaptation does not per se generate resistance to NAIs [47]. Should however NAI resistance mutation(s) already be present in the NA of such a virus a pandemic situation would be difficult to handle as the primary treatment option using stockpiled oseltamivir [2] would fail. Especially during the first phase of a pandemic the lack of treatment options would be troublesome as mass-production of effective vaccines takes several months to accomplish [48], [49]. During the 2009 pandemic, ZA was used alone or in combination with oseltamivir to treat particularly ill patients, e.g. in intensive care units [50], [51]. Despite the low increase in IC_50_ to ZA (<10-fold) by the R292K mutation, it still raises concern as it indicates that overuse of one NAI might harm also other members of this drug class.

In conclusion, we here demonstrate resistance development in an influenza A virus from the N2 group of NAs (H6N2) when one of its natural hosts was infected and exposed to 12 µg/L of OC in their water environment. Drug sensitivity was decreased ≈13,000-fold for OC and ≈7.6-fold for ZA. The mutated virus showed signs of decreased fitness regarding NA-activity and replicative capacity in embryonated hen eggs but infected mallards and transmitted among them as readily as the wild-type virus. Persistence of the mutation through the egg propagation in samples from the later part of the experiment, as well as normalization of viral shedding during the last part of the high concentration experiment might reflect an adaptation of the virus to harbor the mutation. It is possible that the additional mutations D113N or D141N in NA and/or E216K in HA might have influenced such an adaptation, like permissive mutations have been demonstrated in N1 virus that can compensate for the resistance mutation H274Y [4], [5], [22]. Further investigations are needed in this regard, as well as whether the R292K mutation can be retained in mallards also without drug pressure.

Overuse of oseltamivir can result in significant levels in the environment which could lead to resistance development in different avian influenza A viruses with pandemic potential. Thus, an improved sewage treatment and a prudent use of neuraminidase inhibitors are important priorities.

## Materials and Methods

### Virus and Drugs

The influenza A/*Mallard*/Sweden/50908/2006(H6N2) virus was isolated from a wild mallard (*Anas platyrhynchos*) during long-term influenza surveillance studies at Ottenby Bird Observatory in South-East Sweden [52]. The virus was isolated in specific pathogen-free embryonated hens’ eggs according to standard WHO methods [53] by inoculation of the sample into the allantoic cavity of 11-day old embryonated eggs. The allantoic fluid was harvested after 2 days, and influenza A virus was detected by a hemagglutination inhibition assay with chicken erythrocytes. If the HA inhibition assay was negative a second passage was done. The HA subtype was further characterized with subtype-specific hyper-immune rabbit antisera and chicken erythrocytes. The NA subtype was characterized by RT-PCR and sequencing using primers specific for noncoding conserved regions 54,55. The viral concentration of the stock solution used for inoculating the ducks was determined by 50% Embryo Infectious Dose (EID_50_) as previously described [28] to a titer of 10^9.5^ EID_50_/mL. The NA gene was sequenced (in the same way as experimental samples, see below) and deposited to Genbank with accession number JX912288. No known resistance mutations were found.

OC was obtained from the manufacturer F. Hoffmann - La Roche Ltd, Basel, Switzerland. ZA was purchased from the pharmacy as Relenza®. Both compounds were dissolved in double-distilled water to stock solutions and stored at −20°C.

### Experimental Mallard Model

The mallard model is previously described in [28]. In brief, male mallards (*Anas platyrhynchos*), aged 3 to 6 months, were purchased from a Swedish game farm. The ducks were kept in accordance with recommendations from the Swedish Agricultural Board after ethical approval by the Ethical Committee on Animal Experiments in Uppsala (permit C201/11). The uninfected birds were kept in a separate building to minimize the contamination risk. All birds were tested for present or previous influenza A infection by serology (Avian Influenza Virus Antibody test Kit, IDEXX Laboratories Europe, The Netherlands) and RRT-PCR of the influenza A matrix gene from fecal samples [56] before entering the study and the animal house facilities. Each experiment consisted of four generations of mallards, two individuals in each, that each spent 5 days in the experimental room. The first generation of mallards was inoculated by esophageal administration (day zero) of 1 mL viral stock solution, corresponding to 10^9.5^ EID_50,_ and put in the experimental room. In the room they had one sole water source (170L), for swimming and drinking that contained OC and access to feed *ad libitum*. Every third day the next two birds were introduced to the experimental room and spent two days with the previous ones to allow the virus to be fecal-orally transmitted. Each experiment thus lasted 14 days (Figure 1) during which the OC exposure was constant. The water was changed every day and OC was added to concentrations of 120 ng/L (0.42 nM), 1.2 µg/L (4.2 nM) or 12 µg/L (42 nM) in three different experiments.

### Sampling and Analysis

Fecal samples were collected from each individual every day. The birds were put in cardboard boxes from which fresh feces was sampled. If no defecation occurred in the box, cloacal swabbing was performed. The samples were analyzed with RRT-PCR and the NA gene was sequenced. Phenotypic resistance was tested in a neuraminidase inhibition assay. Necropsies and subsequent histopathologic evaluations were performed on two birds in each experiment 5 days pi; one artificially inoculated and one fecal-orally infected. Water samples were collected daily for viral detection and OC concentration analysis.

### Viral Detection and Sequencing of the NA and HA Genes

Viral RNA was isolated using the Magnatrix 8000 extraction robot (Magnetic Biosolutions, Stockholm, Sweden) and Vet Viral NA kit (NorDiag ASA, Oslo, Norway). A one step real-time reverse transcriptase (RRT) PCR targeting the matrix gene of influenza A virus [56] was used to evaluate viral shedding. Reactions of 25 µL were run in a Corbett Research Rotor-Gene 2000 Real-time Thermo Cycler (Corbett Research, Mortlake, Australia) each containing 1 µL RNA-extract, 12.5 µL iScript buffer 2×, 0.5 µL RT-enzyme, 1 µL forward primer, 1 µL reverse primer, 0.3 µL probe in a and 8.7 µL nuclease free H_2_O rendering final concentrations of 400 nM of each primer and 120 nM of probe. Four 10-fold dilution steps of cDNA were included in all RRT-PCR assays. The standard deviations of the CT-values of the cDNA samples ranged from 0.7 to 1.7. Given this small variation, we chose to show RRT-PCR results as CT-values for simplicity.

Two forward and two reverse primers for amplification and sequencing of the NA-gene were designed and purchased from Thermo Hybaid, Interactiva Division (Ulm, Germany) (Table 2). Using these primers, a one-step reverse transcriptase PCR was performed on all samples positive in the RRT-PCR. The PCR had a reaction volume of 25 µL with 5 µL RNA, 12.5 µL reaction buffer, 1 µL H6N2-NA-FW1, 1 µL H6N2-NA-Rev1, 1 µL Superscript III Taq Platinum HiFi RT-enzyme and 4.5 µL nuclease free H_2_O rendering 500 nM final concentrations of primers. The PCR products were confirmed with gel electrophoresis and before sequencing they were purified with ExoSAP-IT (Affymetrix Inc, California, USA) using 2 µL ExoSAP-IT to 24 µL PCR-product. The PCR products were sent to Macrogen Inc. (South Korea or Europe) for sequencing (standard automated Sanger sequencing) of the NA gene using two forward (H6N2-NA-FW1 and H6N2-NA-FW2A) and two reverse (H6N2-NA-Rev1 and H6N2-NA-Rev2aB) primers (Table 2). The sequencing results were analyzed using SeqScape v2.7 software (Applied Biosystems), using the A/*Mallard*/Sweden/50908/2006(H6N2) NA sequence as a reference for detection of new mutations. N2 numbering was used for definition of amino acid residues.

**Table 2:** Primers Used to Amplify and Sequence the NA Gene of A/*Mallard*/Sweden/50908/2006(H6N2).

Primer	Sequence (5′–3′)	Location
H6N2-NA-FW1	TGAACCCAAATCAGAAGATAATAACA	2–27
H6N2-NA-Rev1	GCGAAAGCTTATATAGGCATGAA	1395–1419
H6N2-NA-FW2	GTGTGCATAGCATGGTCCAG	520–540
H6N2-NA-Rev2a	AACCTGAGCGTGAATCCTTG	1100–1120

Sequencing of the HA gene was performed on 8 selected samples, among which 6 were evaluable, from the 12 µg/L experiment that were NA sequenced as wild type, R292K, R292K and D113N, R292K and D141N prior to and after egg propagation. Two forward and two reversed primers were designed to amplify and sequence the HA gene in two segments (Table 3). QIAGEN One-Step RT-PCT Kit (QIAGEN) was used. The reaction volume was 25 µL with 5 µL template, 5 µL reaction buffer, 1 µL H6-HA-FW1 or H6-HA-FW2, 1 µL H6-HA-Rev1 or H6-HA-Rev2, 1 µL RT-enzyme, 1 µL RNA guard, 1 µL DNTPs and 10 µL nuclease free H_2_O rendering 500 nM final concentrations of primers. The PCR product was further confirmed by gel electrophoresis, purified, sequenced and analyzed similar to the NA gene analysis using the A/*Mallard*/Sweden/50908/2006(H6N2) HA sequence as a reference. H3 numbering was used for definition of amino acid residues.

**Table 3:** Primers Used to Amplify and Sequence the HA Gene of A/*Mallard*/Sweden/50908/2006(H6N2).

Primer	Sequence (5′–3′)	Location
H6-HA-FW1	ATTGCAATCATTGTAATAGCGATACTGGC	21–49
H6-HA-Rev1	AGTCTGGCATGTGGCATCGCAGTTCTCG	884–911
H6-HA-FW2	TGAATGTGGAATCTAATGGAAATCTAATCG	784–813
H6-HA-Rev2	TGCATTGCATTGAACCATTTGAACACATC	1676–1704

### Neuraminidase Activity and Inhibition Assay

Viral samples were propagated by inoculation of the fecal sample or first passage isolate into the allantoic cavity of 11-days old embryonated eggs (2 eggs per sample) as described above (under *Virus and Drugs*). Successfully propagated samples were NA re-sequenced prior to the phenotypic resistance assay.

Neuraminidase activity was determined in 96 well plates using the fluorogenic substrate 29-(4-methylumbelliferyl)-a-D-N-acetylneuraminic acid (MUNANA; Sigma) as previously described [57], [58]. The fluorescent product was detected in a GloMax®-Multi Microplate Multimode Reader (Promega) with excitation and emission wavelengths of 365 and 460 nm respectively, measured as relative fluorescent units (RFU). Inhibition by OC and ZA was analyzed in the same MUNANA assay. Serially diluted OC and ZA (to assay concentrations 0.015 to 100,000 nM for OC and 0.015 to 4,000 nM for ZA) were added to duplicates of viral samples. The viral samples were diluted to concentrations corresponding to the log phase of the previously determined NA-activity. All R292K mutants showed relatively low NA activity and were diluted 1∶2 while wild type virus samples were further diluted. Detection of NA inhibition was performed in the same way as the NA activity [57], [58]. Analysis of NA activity and inhibition with determination of IC_50_ was done using Prism5 GraphPad software (GraphPad).

### Statistical Analysis

Hypothesis testing of equality of IC_50_ results and NA activity of mutant to wild type isolates was done with Mann-Whitney non-parametric U test, due to violation of the equal variance assumption of the OC results. 95% confidence intervals of the medians were calculated for each wild type and mutant groups. Statistica12 (StatSoft®) software was used to compute the hypothesis testing.

### Histopathology and Immunohistochemistry

All birds were euthanized with intravenous injection of 100 mg/kg sodium pentobarbital (Pentobarbital vet., 100 mg/ml). Necropsies were performed on 6 birds in total. Samples from inner organs and 10 levels of the GI tract were fixed in 10% neutral buffered formalin, routinely processed and stained for evaluation of histopathology and presence of intracellular virus by immunohistochemistry (IHC) staining with an anti-influenza A nucleoprotein (NP) monoclonal antibody (HB65, EVL, The Netherlands) [59] as described in detail in [29].

### Analysis of OC Concentration in the Water

Water samples were collected each day after 24 h exposure (when the water was changed). To estimate the loss of OC during the 24 h, water samples were also collected directly after addition of OC to the water. An on-line solid phase liquid extraction/liquid chromatography-tandem mass-spectrometry (SPE/LC-MS/MS) system was used to analyze the OC levels in the water samples. The SPE/LC-MS/MS system used has been described in detail previously in [28] with the exception that an extraction Oasis HLB column (2.1×20 mm, 15 µm particle size (Waters S.A.S., Saint-Quentin, En Yvelines Cedex, France)) was used in this system. Briefly, 10 mL sample (for the highest exposure level a sample/water volume of 1/10 was used) was pre-filtered (45 µm Filtropur) and acidified (0.1% of formic acid on volume basis) and thereafter 1 ml sample was analyzed using the SPE/LC-MS/MS system. Samples were quantified using internal standard method (deuterated OC obtained from Roche, F. Hoffmann-La Roche Ltd, Basel, Switzerland was used as internal standard) with six calibration points.

### NCBI Database Screening

The NA sequences of all avian influenza A/N2 viruses available in the NCBI Influenza Virus Sequence Database on April 19^th^, 2012 were aligned and analyzed regarding mutations at amino acid positions 292, 113 and 141 using the BioEdit v7.0.5.3 software.
